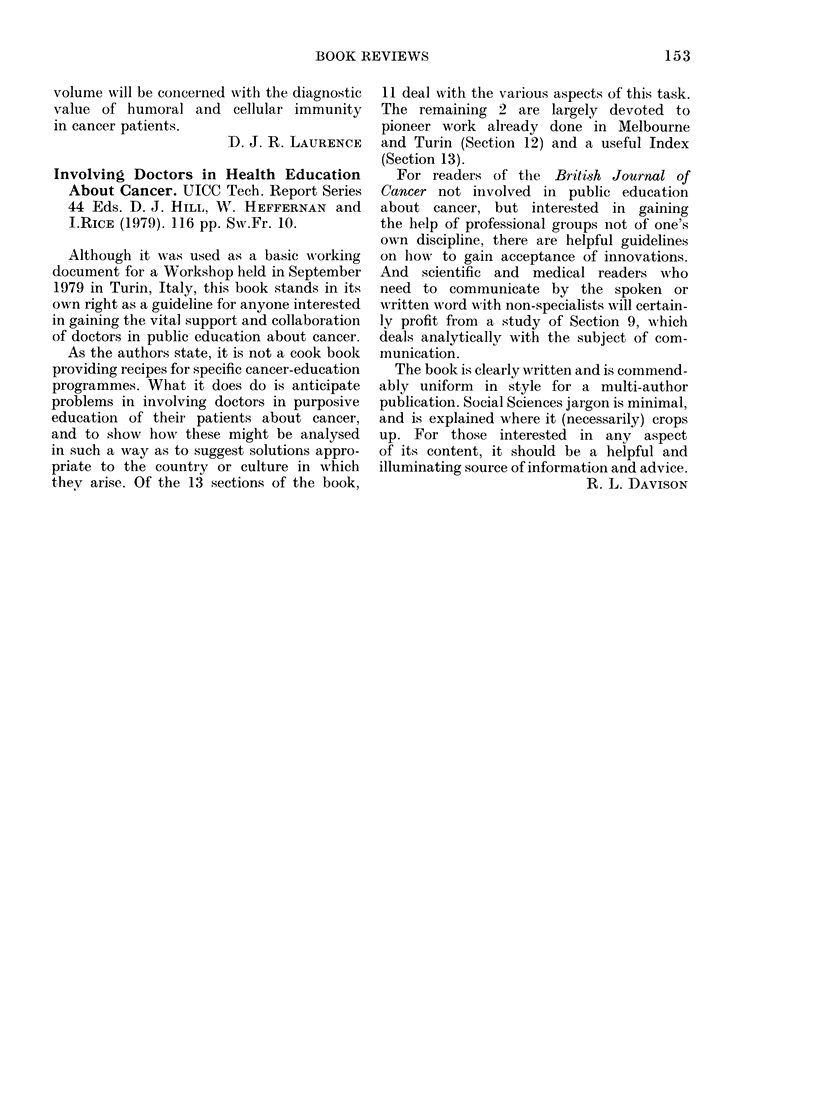# Involving Doctors in Health Education About Cancer

**Published:** 1980-01

**Authors:** R. L. Davison


**Involving Doctors in Health Education About Cancer.** UICC Tech. Report Series 44 Eds. D. J. HILL, W. HEFFERNAN and I.RICE (1979). 116 pp. Sw.Fr. 10.

Although it was used as a basic working document for a Workshop held in September 1979 in Turin, Italy, this book stands in its own right as a guideline for anyone interested in gaining the vital support and collaboration of doctors in public education about cancer.

As the authors state, it is not a cook book providing recipes for specific cancer-education programmes. What it does do is anticipate problems in involving doctors in purposive education of their patients about cancer, and to show how these might be analysed in such a way as to suggest solutions appropriate to the country or culture in which they arise. Of the 13 sections of the book, 11 deal with the various aspects of this task. The remaining 2 are largely devoted to pioneer work already done in Melbourne and Turin (Section 12) and a useful Index (Section 13).

For readers of the *British Journal of Cancer* not involved in public education about cancer, but interested in gaining the help of professional groups not of one's own discipline, there are helpful guidelines on how to gain acceptance of innovations. And scientific and medical readers who need to communicate by the spoken or written word with non-specialists will certainly profit from a study of Section 9, which deals analytically with the subject of communication.

The book is clearly written and is commendably uniform in style for a multi-author publication. Social Sciences jargon is minimal, and is explained where it (necessarily) crops up. For those interested in any aspect of its content, it should be a helpful and illuminating source of information and advice.

R. L. DAVISON